# 

**Published:** 1890-06

**Authors:** 


### BOOKS AND PAMPHLETS RECEIVED.

Estatutos de la Sociedad, Estudios Preparatorios de Medicina. Republica de Costa Rica. 1890.

"The Blunt Curette in Uterine Hemorrhage." By T. W. Kay, M.D. Reprinted from *New York Medical Journal.*

Eighteenth Annual Report of the Zoological Society of Philadelphia. 1890.

Annual Report of the American Museum of Natural History. New York, '90.
Johns Hopkins University Circulars. Baltimore.
Bulletin de la Société Zoologique de France. Paris.
Journal Bombay Natural History Society. Vol. IV, Nos. 3 and 4.
Bulletin de L'Accademie Royal de Medecine de Belgique. Bruxelles.
Bulletin Mensuel Société, Linneenne du Nord de la France. Amiens.
Der Zoologische Garten. Frankfurt-a-M.
Bollettino Scientifico. Pavia.
The Canadian Record of Science. Montreal.
The Proceedings of the Linnean Society of New South Wales. Vol. IV, Pl. 2. Sidney.

Veterinary Dental Surgery. By T. D. Hinabauch, M.S., V.S., Professor of Veterinary Science of Purdue University, Lafayette, Ind. Illustrated. Published by the author. Lafayette, Ind. 1889.

This book of 256 pages, illustrated with cuts of the form and structure of the teeth, pathologic conditions and instruments used in dental surgery, is a useful addition to our list of text-books for veterinary practitioners and students. Several chapters, some of them by other writers than the author, on "Dental Neuralgia," "Filling of Horses' Teeth," and other subjects in veterinary medicine, are beyond the practical conception of the average veterinarian, but on the whole the book will be found interesting and valuable.

Bericht uber die VI Plenar-Versammlung des Deutschen Veterinärrathes, am 17 und 18 June, 1889, zu Eisenach. Karlsruhe, 1889.

The account of this meeting under the presidency of Dr. Lydtin, which was attended by government and college representatives from Baden, Bavaria, Brunswick, Mechlenburg, Oldenburg, Prussia, Saxony, Thuringia and Wurtemburg furnishes most valuable papers concerning the local and sanitary status of the veterinary profession in Germany, and can be consulted with benefit by those interested in State legislation.

Sixteenth Annual Report of the State Board of Health of the State of Michigan for the Fiscal Year Ending June 30th, 1888. Lansing, Mich., 1889. Consumption, hydrophobia, glanders, lump-jaw in cattle, and poisons in dairy products are among the subjects treated of interest to veterinarians.

"Ætiology of Tuberculosis." Dr. R. Koch. Translated by Rev. T. Saure.

Transactions of the Massachusetts Veterinary Medical Association, reprinted from the *American Veterinary Review*. Wm. R. Jenkins, New York. 1890. A valuable work of reference of ninety-seven pages now published in convenient book form.

Department of Agriculture, Washington, 1890. Report No. 71. Distribution and Consumption of corn and wheat. Report No. 72. Condition of Winter grain and the condition of farm animals. Report on Flax, Hemp, Ramie and Jute. Report on the Beef Supply of the U. S.

Journal of the Asiatic Society of Bengal. Vol. LVIII, Part II, No. 2, 1889; Supplement No. 2, Calcutta, 1889; Part II, No. 3, 1889; Part II, No. 4, 1889; Part II, Supplement No. 1, 1890.

